# The oculomotor microcosm

**DOI:** 10.1093/braincomms/fcae337

**Published:** 2024-10-01

**Authors:** Chrystalina A Antoniades

**Affiliations:** Nuffield Department of Clinical Neurosciences, John Radcliffe Hospital, University of Oxford, Oxford, OX3 9DU, UK

## Abstract

This scientific commentary refers to ‘Noradrenergic modulation of saccades in Parkinson’s disease’, by  Orlando *et al*. (https://doi.org/10.1093/braincomms/fcae297).

This scientific commentary refers to ‘Noradrenergic modulation of saccades in Parkinson’s disease’, by  Orlando *et al*. (https://doi.org/10.1093/braincomms/fcae297).

The neurophysiology and neuroanatomy of saccades, the most common type of eye movements we all make, have been well characterized over the last few decades (for a comprehensive overview, see Leigh and Zee’s^1^*The Neurology of Eye Movements*). Saccades have also been studied extensively in neurodegenerative conditions such as Parkinson’s disease, which causes a characteristic set of oculomotor abnormalities. One specific area of interest is how the various classes of medication used in such conditions can significantly alter some of the well-established saccadic parameters such as speed and accuracy associated with both reflexive and higher order oculomotor tasks. Dopaminergic medication is the mainstay of treatment for the control of motor symptoms associated with Parkinson’s disease, and previous work has studied the effects of dopaminergic drugs on saccades. Our group and others^2-4^ have shown that prosaccadic (reflexive) latency is significantly affected by dopaminergic medication. Interestingly, antisaccadic (looking to the opposite direction) latency remains unaffected by medication despite exhibiting a large disease effect.

Parkinson’s disease presents with a wide range of symptoms, from the well-known motor issues such as tremor to the often less well-recognized non-motor symptoms. Much of the research in Parkinson’s disease, and all of the effective symptomatic treatments, have focused on the motor part of the disorder. Other aspects of the condition such as cognitive impairment can be just as disabling, but effective treatment of this has been more elusive. One approach has looked into targeting the deficits present in noradrenergic transmission.^5^ The noradrenergic neurons of the locus coeruleus in the brainstem, firing at a slow rate during waking, are the primary source of forebrain noradrenaline, and the locus coeruleus is one of the early sites of Parkinson’s disease pathology, leading to the idea that the noradrenergic system might be a potential target for therapeutic intervention.

Atomoxetine, a noradrenaline reuptake inhibitor, is a drug that has been shown to improve behavioural performance in Parkinson’s disease.^6^ Orlando *et al.*,^7^ in their recent article in *Brain Communications*, have studied the noradrenergic modulation of saccades in a group of people with Parkinson’s along with a matched control group, hypothesizing that there would be a change in oculomotor control in people with Parkinson’s after administration of atomoxetine. To investigate this, they used the well-known prosaccadic and antisaccadic tasks.^8^ This was a double-blind, placebo-controlled, crossover study in which the participants received 40 mg of atomoxetine on one occasion and placebo on another occasion, with the order being randomized. Venepuncture was conducted 2 h after the placebo or drug administration, and then participants completed the oculomotor battery. The sessions were at least 6 days apart and were completed at similar times of the day while patients were kept on their regular anti-parkinsonian medications.

This is an interesting study highlighting the changes in saccadic behaviour that such a drug can bring about. Prosaccades became faster, and antisaccadic errors increased in the Parkinson’s disease patients when on atomoxetine relative to placebo; the higher rates of antisaccadic errors were associated with faster prosaccadic latencies, a known form of speed–accuracy tradeoff. Saccade studies are a nice way to quantify *changes* like this, free from potentially confounding limb motor effects due to symptoms such as tremor, bradykinesia and rigidity.

This study by Orlando *et al.* paves the way for using saccadic eye movement studies to understand the effects of noradrenergic treatments. Since atomoxetine can have an onset of action within 1–2 weeks of starting treatment with a trajectory of response that is incremental over a longer period of time,^9^ it would be interesting (of course demanding to do) to study the effects on saccades of different doses at various time points in Parkinson’s disease. Establishing standardized criteria on the optimal dose and timeline may open up the use of atomoxetine to a wider number of people with Parkinson’s, especially those that might benefit from its use at the earlier stages of this disorder, and thus positively influence the quality of life of these individuals.

Thinking of the wider use of atomoxetine and what data are out there to help us understand its impact on the oculomotor system, it is important to mention that it is a drug that is primarily used for treating children and adults with attention deficit hyperactivity disorder. Research in this condition suggests that eye tracking may be useful in diagnosis,^10^ and by extension, it is tempting to speculate that measurement of saccades (saccadometry) might be helpful in monitoring treatment.

Understanding pharmacological interactions with eye movements is important, not only for enhancing the direct treatment of neurological or psychiatric conditions but also for understanding how certain drugs may be used to target the non-motor side of disorders using the oculomotor system. Saccades, in particular, have proven invaluable because they eliminate the motor component, which is well known to interfere with performance during tasks. As my late mentor Professor Roger Carpenter wrote: ‘The saccadic system is a neurological microcosm. The inputs and outputs of the oculomotor system are circumscribed and easy to measure and allow us to regard it quantitatively in a way that is scarcely possible elsewhere: in many cases, a few quick measurements of eye movements can provide as accurate a neurological diagnosis as a brain scan’.^11^
